# β-FeOOH mediated heavy indium doping to release the tunnel space in α-MnO_2_

**DOI:** 10.1039/d6ra07481b

**Published:** 2026-11-06

**Authors:** Hongmei Zhao, Song Li, Jing Wang, Wai-Yeung Wong, Pengfei Liu, Jing Li, Junjie He

**Affiliations:** a Department of Science and Technology, Yunnan Agricultural University Kunming 650201 China junjiehe@ynau.edu.cn lijing69@ynau.edu.cn; b Department of Applied Biology and Chemical Technology, Research Institute for Smart Energy, The Hong Kong Polytechnic University Hung Hom Hong Kong China wai-yeung.wong@polyu.edu.hk; c Spallation Neutron Source Science Center, Institute of High Energy Physics, Chinese Academy of Sciences Dongguan 523803 China

## Abstract

Alpha manganese dioxide (α-MnO_2_) has shown promising applications in energy storage, electrocatalysis, ion adsorption/separation due to its unique tunnel structure. In the preparation of α-MnO_2_, the potassium ion template is important for inducing the formation of the tunnels, but it leaves a large amount of potassium ions inside the final α-MnO_2_ tunnel because of the strong interaction between potassium ion and the inner wall of MnO_2_ tunnels. The potassium ion not only occupies and wastes the space of the tunnel, but also hinders the transport of the ions in the tunnels. Here, with the assistance of iron, indium is introduced into α-MnO_2_. The ICP-MS results confirmed that only 0.31% of In ions is doped in α-MnO_2_ without Fe and the content of In is significantly increased to 0.81% when Fe is added as an auxiliary species. Theoretical calculation reveals the binding energy between Fe–In co-doped α-MnO_2_ and potassium ions is significantly decreased. Moreover, the conductivity is increased from 0.177 µS cm^−1^ to 1.55 µS cm^−1^. Based on these improvements, the FeInMnO_2_ cathode was fabricated and presented excellent charge storage performance in zinc ion battery.

## Abstract 2

This study introduced Fe-assisted heavy-In doping into α-MnO_2_ to yield FeInMnO_2_: In content rose from 0.31% to 0.81% and conductivity from 0.177 to 1.55 µS cm^−1^. The cathode presented excellent charge storage in aqueous Zn-ion batteries.

## Graphical abstract

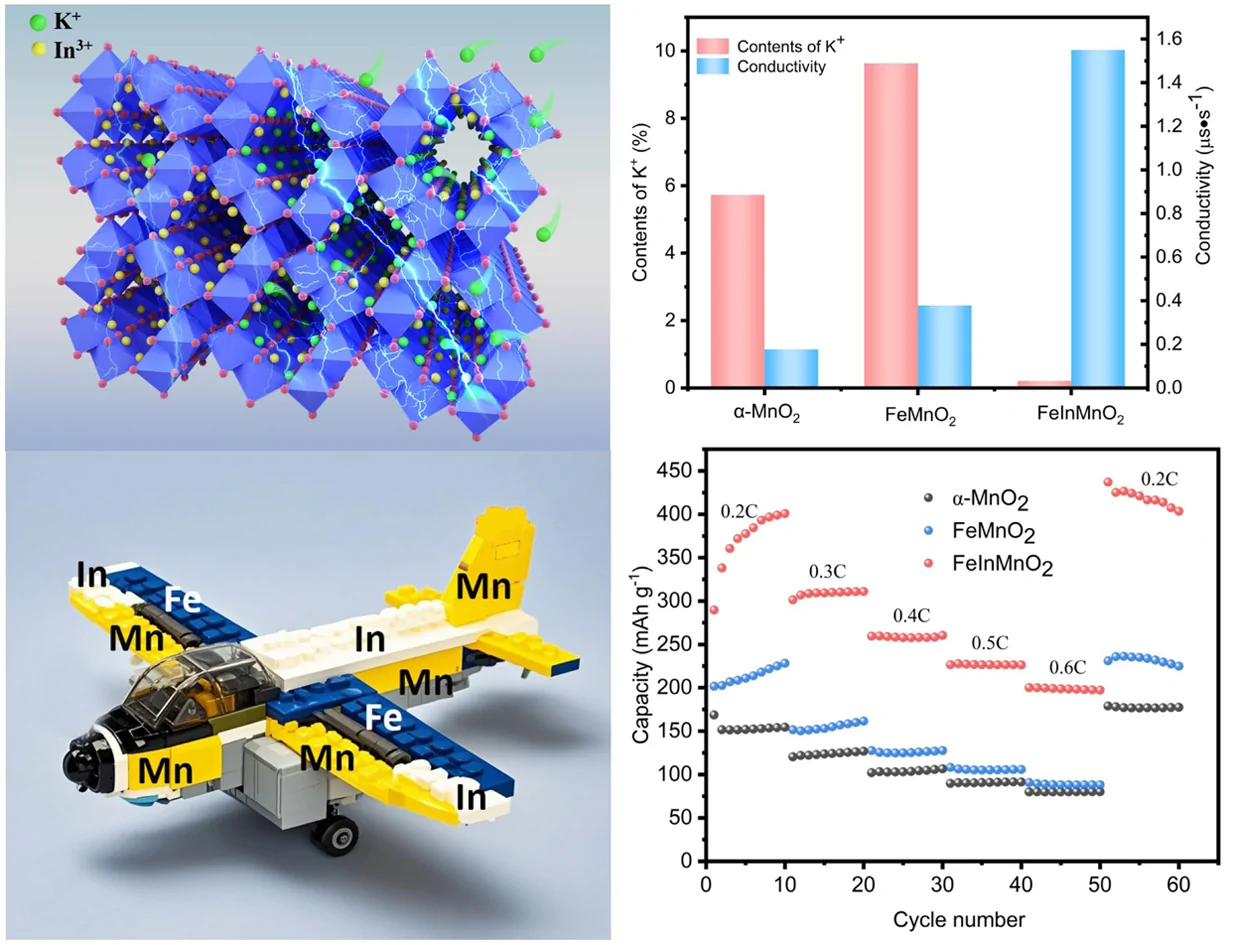

## 1 Introduction

Due to its varied polycrystalline structures, low cost, environmental friendliness, and suitable pore size, α-MnO_2_ is widely used, especially in energy storage and electrocatalysis,^1,2^ ion adsorption/separation,^3,4^ biological applications,^5^ and wastewater remediation.^6^ These applications are closely related to its excellent and efficient tunnel structures. Different pore or tunnel structures support selective entry and transfer of different ions, exhibiting excellent cation exchange properties.^7^ In addition, the high surface area and abundant surface oxygen resulting from the porous structure are the root causes of its high activity.^8^ α-MnO_2_ has an open tunnel structure which can provide sufficient crystallographic volume. Furthermore, changes in synthesis conditions and stable ions provide controllability over the specific size, shape, and ion content of the structural tunnel, exhibiting a certain degree of adjustability.^9^ In the synthesis process of α-MnO_2_, K^+^ is often used as the template to control the pore structure and the formation of crystal phase. But the template also ultimately remains in the tunnel pores of α-MnO_2_, occupying the volume inside the tunnel, covering the active sites, and hindering ion transport. Moreover, their binding effect is strong and difficult to remove through simple methods.^10^ It is worth noting that when K^+^ template ion will make α-MnO_2_ electrochemically inert.^11,12^ This is related to the fact that K^+^ occupies the reactive sites of α-MnO_2_, and its strong binding energy with oxygen atoms in MnO_2_ causes the corresponding sites to lose their energy storage activity. Therefore, releasing these K^+^ ions is a key to increase the available pore volume of tunnels, increase the number of catalytic active sites, and enhance the energy storage capacity.^13^ However, there have been no reports on how to reduce the binding force between O atoms and K^+^ ions in α-MnO_2_ to remove dead adsorbed potassium ions. By introducing atoms with high electron affinity, it is expected to reduce the electron cloud density on the surface O atoms of α-MnO_2_ and their binding energy with K^+^ ions.^14^ However, it is difficult to perform substitutional doping of manganese with these metal elements because of their inconsistent coordination numbers, low tolerance of α-MnO_2_ to dopant atoms, limited doping concentration and achieving ideal results.^15^ Therefore, how to perform high concentration substitutional doping on the Mn atoms is a worthwhile research question.

In response to this, this study used β-FeOOH with the same crystal topology as α-MnO_2_ to mediate high concentration In doping modification of α-MnO_2_ (referred to as FeInMnO_2_). It greatly reduces the binding energy between α-MnO_2_ and K^+^ ions and releases energy storage active sites to obtain a cathode material with excellent electrochemical performances.

## 2 Experimental section

### 2.1 Preparation of α-MnO_2_, FeMnO_2_ and FeInMnO_2_

#### 2.1.1 α-MnO_2_

α-MnO_2_ were synthesized by a hydrothermal method^16^ 0.003 M MnSO_4_·H_2_O (from Shanghai Macklin Biochemical Co., 99.9%) and 2 mL 0.5 M H_2_SO_4_ were added to 90 mL water to prepare an aqueous solution A. An solution denoted as B was prepared by dissolving 0.1 M KMnO_4_ (from Chongqing Chuangdong Chemical Co., AR) in 20 mL aqueous solution B was added drop-wise into solution A under continuous stirring at room temperature for 2 h. Then the mixture was transferred to stainless steel hydrothermal kettles and heated at 120 °C for 12 h. After cooling, a dark brown precipitate was collected by centrifugation, washed several times with water, and finally dried at 80 °C.

#### 2.1.2 FeMnO_2_

FeMnO_2_ were prepared as follows 0.003 M MnSO_4_·H_2_O (Shanghai Macklin Biochemical Co., 99.9%), 2 mL 0.5 M H_2_SO_4_ and 5 mol% FeCl_3_ (Shanghai Macklin Biochemical Co., 99%) of the total metal ions were added to 90 mL water to prepare an aqueous solution A. An solution denoted as B was prepared by dissolving 0.1 M KMnO_4_ in 20 mL aqueous solution. B was added drop-wise into solution A under continuous stirring at room temperature for 2 h. Then the mixture was transferred to stainless steel hydrothermal kettles and heated at 120 °C for 12 h. After cooling, a dark brown precipitate was collected by centrifugation, washed several times with water, and finally dried at 80 °C.^17^

#### 2.1.3 FeInMnO_2_

FeInMnO_2_ were prepared as follows 0.003 M MnSO_4_·H_2_O (Shanghai Macklin Biochemical Co., 99.9%), 2 mL 0.5 M H_2_SO_4_, 5 mol% FeCl_3_ (Macklin Biochemical Co., Shanghai, 99%) of the total metal ions and 0.7 mol% In_2_(SO_4_)_3_ (99.99%) were added to 90 mL water to prepare an aqueous solution A. A solution denoted as B was prepared by dissolving 0.1 M KMnO_4_ (Chongqing Chuangdong Chemical Co., AR) in 20 mL aqueous solution. B was added drop-wise into solution A under continuous stirring at room temperature for 2 h. Then the mixture was transferred to stainless steel hydrothermal kettles and heated at 120 °C for 12 h. After cooling, a dark brown precipitate was collected by centrifugation, washed several times with water, and finally dried at 80 °C.

#### 2.1.4 In-doped β-FeOOH

β-FeOOH were synthesized by a hydrothermal method^18^ 5 mol% FeCl_3_ (Shanghai Macklin Biochemical Co., 99%) was added to 370 mL H_2_O to prepare an queous solution A. Different amounts of In_2_(SO_4_)_3_ (0.3 mol%, 0.7 mol%, 2.5 mol%, 5 mol%) were added to solution A. Then the mixture was transferred to stainless steel hydrothermal kettles and heated at 80 °C for 8 h. After cooling, a precipitate was collected by centrifugation, washed several times with water, and finally dried at 60 °C in an oven.

### 2.2 Materials characterization

SEM images were obtained on a Hitachi Regulus8100 instrument at an acceleration voltage of 15 keV. XRD data were obtained using a Smartlab 9 kW equipped with a Cu Ka radiation (*λ* = 1.54 Å) source. TEM images were obtained on a F200S instrument. HAADF-STEM and energy dispersive X-ray (EDX) mapping spectra were recorded on a FEI Titan Cubed Themis G2 instrument at an acceleration voltage of 300 kV. XPS were recorded on a Thermo Fisher Scientic EsCalab 250Xi spectrometer using an aluminum anode (Al 1486.6 eV) Raman spectra were obtained on a Horiba HR Evolution spectrometer using 532 nm laser excitation. Inductively coupled plasma mass spectrometry (ICP-MS) data were obtained on an Agilent 7700s spectrometer. To evaluate the conductivity of the materials, a specific voltage was applied between the disks, and the current was recorded, while 10 MPa pressure was applied for all samples. Are measurement of the thickness of the sample disks, the resistance and the conductivity of the materials were calculated using Ohm's law.

### 2.3 Electrochemical measurements and quantification

Cyclic voltammetry (CV) curves, GCD curves of the samples were measured on an electrochemical workstation (Tianjin Lanlike/LK 2010). A two-electrode system was used to test the electrochemical performance of the active materials. 2 M ZnSO_4_ (Shanghai Macklin Biochemical Co., 99.995%) + 0.1 M MnSO_4_ solution were used as the electrolyte. Zinc metal foil was used as the anode and α-MnO_2_/nickel was used as cathode. The electrode preparation followed the procedure described in our previous work.^17^ The CV curves have a sweep rate range of 1–20 mV s^−1^.

Surface capacitive or diffusion contribution of three materials were calculated using Dunn's test.^19^ The CV curves were measured at different scan rates, the current density of each curve was described as follows:

1 *i* = *k*_1_*v* + *k*_2_*v*^0.5^

where *v* is the scan rate (V s^−1^). *k*_1_ and *k*_2_ can be analysized according to the CV curves obtained at different scan rates. For any CV curve at specific scan rate, *k*_1_*v* is the capacitive contribution, while *k*_2_*v*^0.5^ is the diffusion contribution.

Long term charge–discharge cycling tests and GITT measurements were conducted on a Neware battery test system. The diffusion coefficient of Zn^2+^ was obtained using eqn (2):2
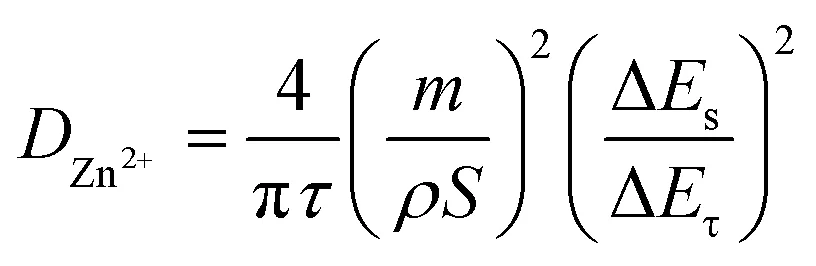
where *τ* is the constant current pulse time. The *m* and *ρ* are the mass and calculated density of α-MnO_2_. The *S* is the electrode–electrolyte interface area. The Δ*E*_s_ is the steady-state voltage change caused by the current pulse. The Δ*E*_τ_ are voltage changes during the constant current pulse.

## 3 Results and discussion

### 3.1 Fabrication and characterization of three materials

Fig. 1a–c shows the scanning electron microscope (SEM) images of α-MnO_2_, 5 mol% Fe-doping (FeMnO_2_), and 0.7 mol% In plus 5 mol% Fe-doping (FeInMnO_2_). A α-MnO_2_ nanofibre structure are observed in all cases.^16^ This suggests that Fe and In dopants will not change the morphology of α-MnO_2_. High-resolution transmission electron microscopy (HRTEM) image of FeInMnO_2_ (Fig. 1d) is also obtained. α-MnO_2_ nanofibres with a lattice distance of 0.239 nm for the (211) crystal plane is resolved.^19^ High-angle annular dark field (HAADF)-STEM (Fig. 2e and f) depicts 2 × 2 tunnels typical for α-MnO_2_ and FeInMnO_2_. Interestingly, the tunnel structures of FeInMnO_2_ exhibit uniform lattice and some of the tunnel radius is broadened, which facilitates the diffusion of ions. STEM-EDS analysis (Fig. 1g–j) shows that the elemental distribution of Mn, Fe, In and K. It confirms that the Fe and In are doped into the crystal structure of α-MnO_2_ rather than the mixture of the metal oxides. The ICP-MS results show that the content of In ions is only 0.31% when In is directly doped in α-MnO_2_ and the content of In is significantly increased to 0.81% (Table 1) when 5 mol% iron is added as an auxiliary species.

**Fig. 1:**
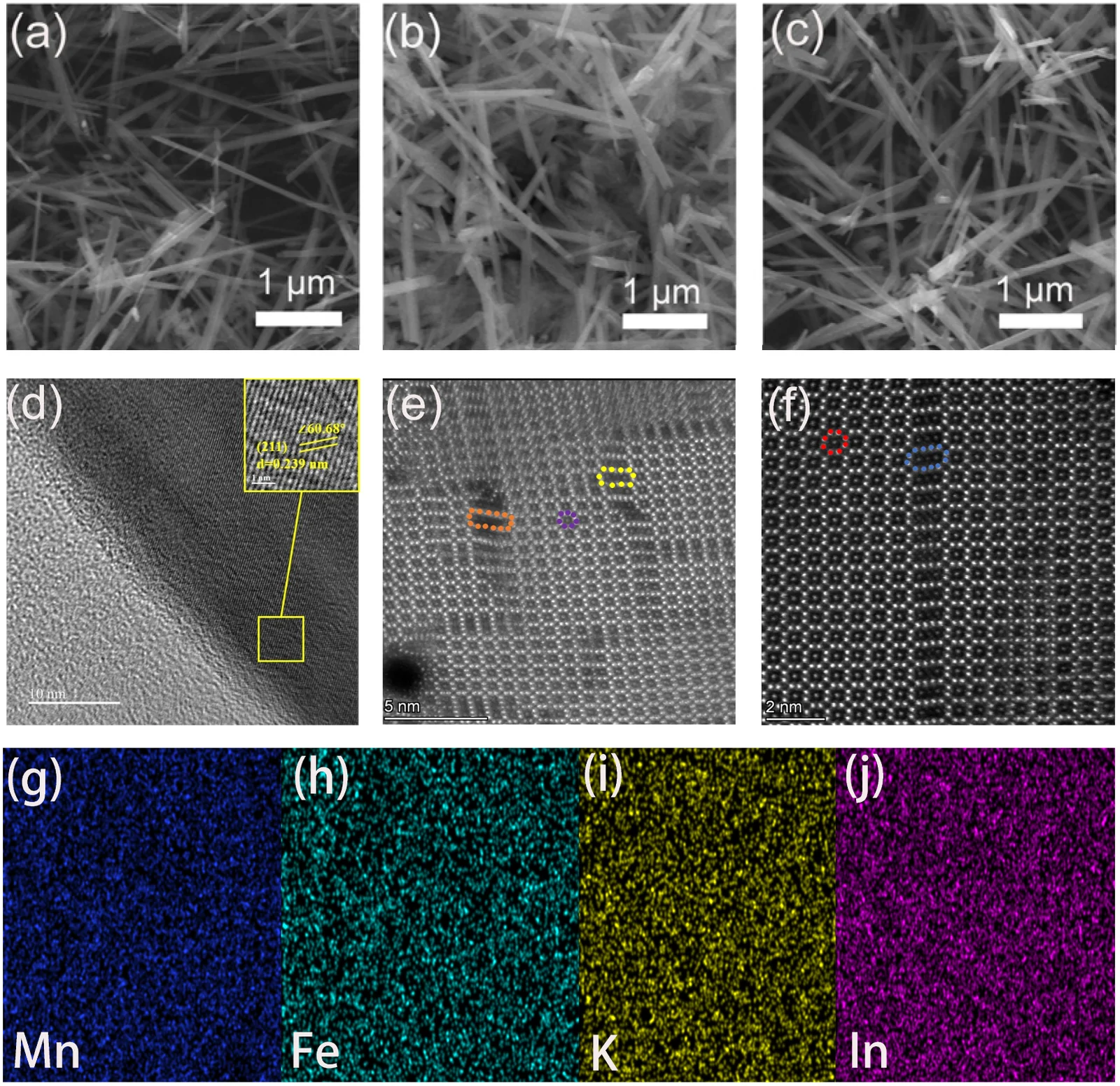
(a–c) SEM images α-MnO_2_, FeMnO_2_, FeInMnO_2_. (d) TEM image of FeInMnO_2_ and the yellow inset shows lattice fringes along the (211) planes. (e and f) HAADF-STEM images of α-MnO_2_ and FeInMnO_2_. Colored circles corresponding to Mn columns (red and purple circle: 2 × 2 tunnels; blue, yellow and orange circles: 2 × 4 tunnels). (g–j) Elemental mapping spectra of Mn, Fe, In and K with the corresponding HAADF-STEM of FeInMnO_2_.

**Fig. 2:**
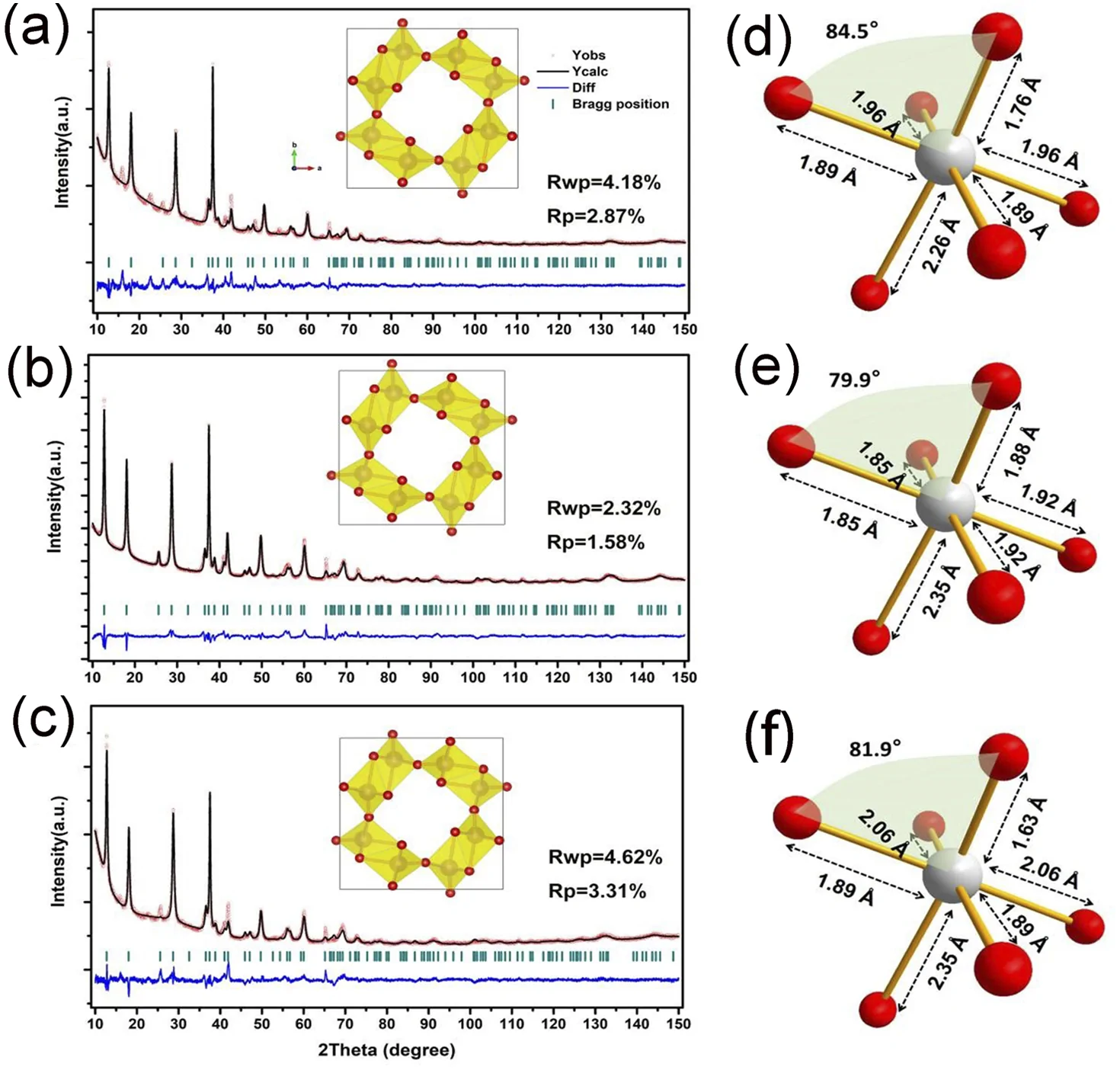
(a–c) The X-ray diffraction patterns and the fit curves from the Rietveld structure (d–f) refinement of α-MnO_2_, FeMnO_2_ and FeInMnO_2_.

**Table 1:** The ICP-MS results of FeInMnO_2_ and In-doped α-MnO_2_

Samples^a^	Element	Concentration/g kg^−1^	Mass/%	Mole fraction (In/Mn)/%
FeInMnO_2_	In	6.21	0.62	0.81
	Mn	366.37	36.63	
In-doped α-MnO_2_	In	1.80	0.18	0.31
	Mn	278.46	27.84	

a 0.7% mol of In was added for both samples.

The crystal phase of FeInMnO_2_ is not changed according to the X-ray diffraction (XRD) data (Fig. 2a–c and S1). All of the samples present sharp diffraction peaks at 12.7°, 18.0°, 28.6° and 37.5°, respectively. Their high crystallinity and pure crystal phase indicate the framework of α-MnO_2_ is retained after Fe/In incorporation. As shown in Fig. 2d–f, only the bond length (2.06 Å) and bond angle of α-MnO_2_ have been changed when 5 mol% Fe and 0.7 mol% are doped into α-MnO_2_. X-Ray photoelectron spectroscopy (XPS) of FeInMnO_2_ is used to analyse the chemical oxidation state of FeInMnO_2_. Fig. 3a illustrates the Mn 2p region including Mn^3+^ and Mn^4+^ states. The spin–orbit doublet of Mn 2p_3/2_ and Mn 2p_1/2_ are found at 642.0 eV and 653.8 eV, which are 11.8 eV apart. This is consistent with the reported values for Mn-based oxides.^20^ The Fe 2p spectra are shown in Fig. 3b. The characteristic Fe spin–orbit peaks of 2p_3/2_ is located at 710.70 eV, and while Fe 2p_1/2_ is located at 724.85 eV, which indicate the existence of a mixture of Fe^2+^ and Fe^3+^.^21^Fig. 3c displays the In 3d spectrum. A perfect fit is found to the two peaks which are located at 444.6 and 452.2 eV and this can be attributed to the characteristic spin–orbit split of 3d_5/2_ and 3d_3/2_, respectively.^22^ This results show that the Indium valency is +3. It can be inferred that In has been successfully doped into α-MnO_2_ with the assistance of Fe. To illustrate the mechanism of the Fe assisted In doping, the XRD of β-FeOOH with different amounts of In are tested (Fig. 3d). No impurities are observed and all peaks of five samples are attributed to akaganeite (JCPDS 34-1266). The characteristic diffraction peaks of β-FeOOH at 11.8° (110), 16.8° (200), 26.8° (310), 34.1° (400) and 35.3° (211) are observed.^23^ These peaks are in good agreement with α-MnO_2_ (JCPDS 44-0141, Fig. S1). When the concentration of In is increased to 5 mol%, the crystalline shape of β-FeOOH did not undergo any significant change and still maintain a good crystalline shape. This means that β-FeOOH has a higher Indium ion tolerance. When In and Fe co-doping strategy was carried out in α-MnO_2_, more In can enter together with Fe into α-MnO_2_ and release more active sites. This is consistent with the results of ICP-MS test and further confirmed by theoretical studies. The specific surface plots (Fig. 3e and S2) also further demonstrate that the specific surface area and pore volume achieved an increase over 50% after Fe and In doping, suggesting that there are superior ion diffusion channels in FeInMnO_2_.

**Fig. 3:**
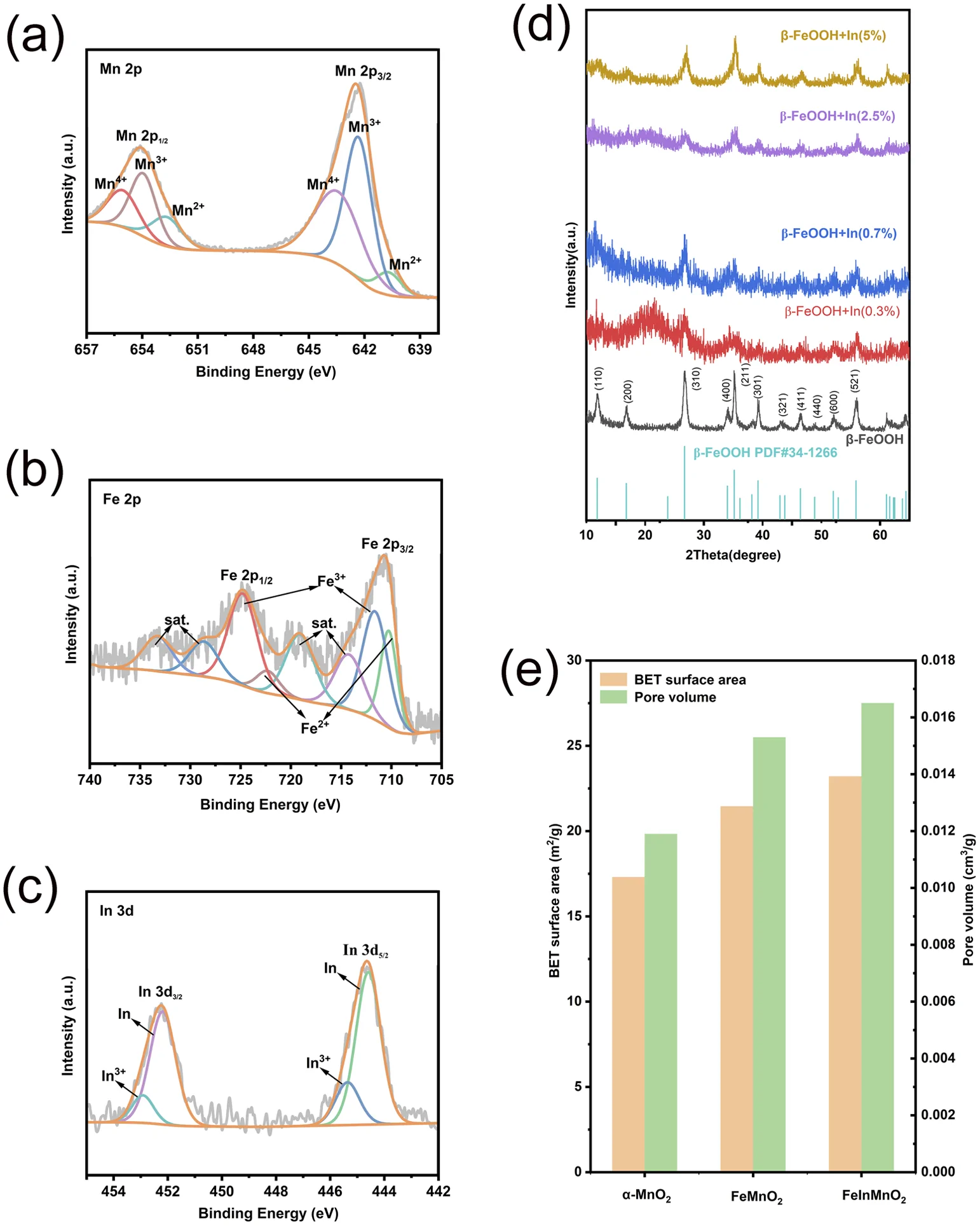
(a–c) The XPS spectral fitting of FeInMnO_2_. (d) The XRD patterns of β-FeOOH with different concentrations of In. (e) The BET and pore size distribution map of α-MnO_2_, FeMnO_2_ and FeInMnO_2_.

To further demonstrate that Fe and In doping releases more active sites, the binding energy was calculated, while the K^+^ content of different materials have been evaluated. We performed first principles calculations to study the binding energy and electronic structure of the three materials (Fig. 4a–d and S3). The binding energy with K^+^ of Fe–In co-doped α-MnO_2_ (FeInMnO_2_) is significantly reduced, while the binding energy with Zn^2+^ is significantly increased. Moreover, the total binding energy of FeInMnO_2_ is also increased. In Fig. 3d, the electron density on Mn atoms is slightly increased after doping. This result illustrates the substitutional doping effect from Fe–In co-doping, in which K^+^ ions are released and the stability of FeInMnO_2_ is enhanced. Following our previously established methodology, uniform-weight solid samples were sandwiched between two stainless steel disks within a custom-designed plastic chamber to enable precise quantification of material electrical conductivity.^17,24^ As shown in Fig. 4e the K^+^ content of the FeInMnO_2_ material is significantly reduced, which frees up the tunneling space. And the conductivity is increased from 0.177 µS cm^−1^ to 1.55 µS cm^−1^. To further investigate the effect of In doping on the crystal structure, the Raman spectra of α-MnO_2_ and FeInMnO_2_ were recorded (Fig. 4f and g). The results shows that the peak located at 600–640 cm^−1^ corresponds to the Mn–O vibration of MnO_6_ octahedra.^17^ Blue shifting of the Fe–In co-doped have occurred, which can be attributed to an increase in the force constant according to Hooke's law. This indicates that Fe–In co-doped is favourable for reinforcing its structure.

**Fig. 4:**
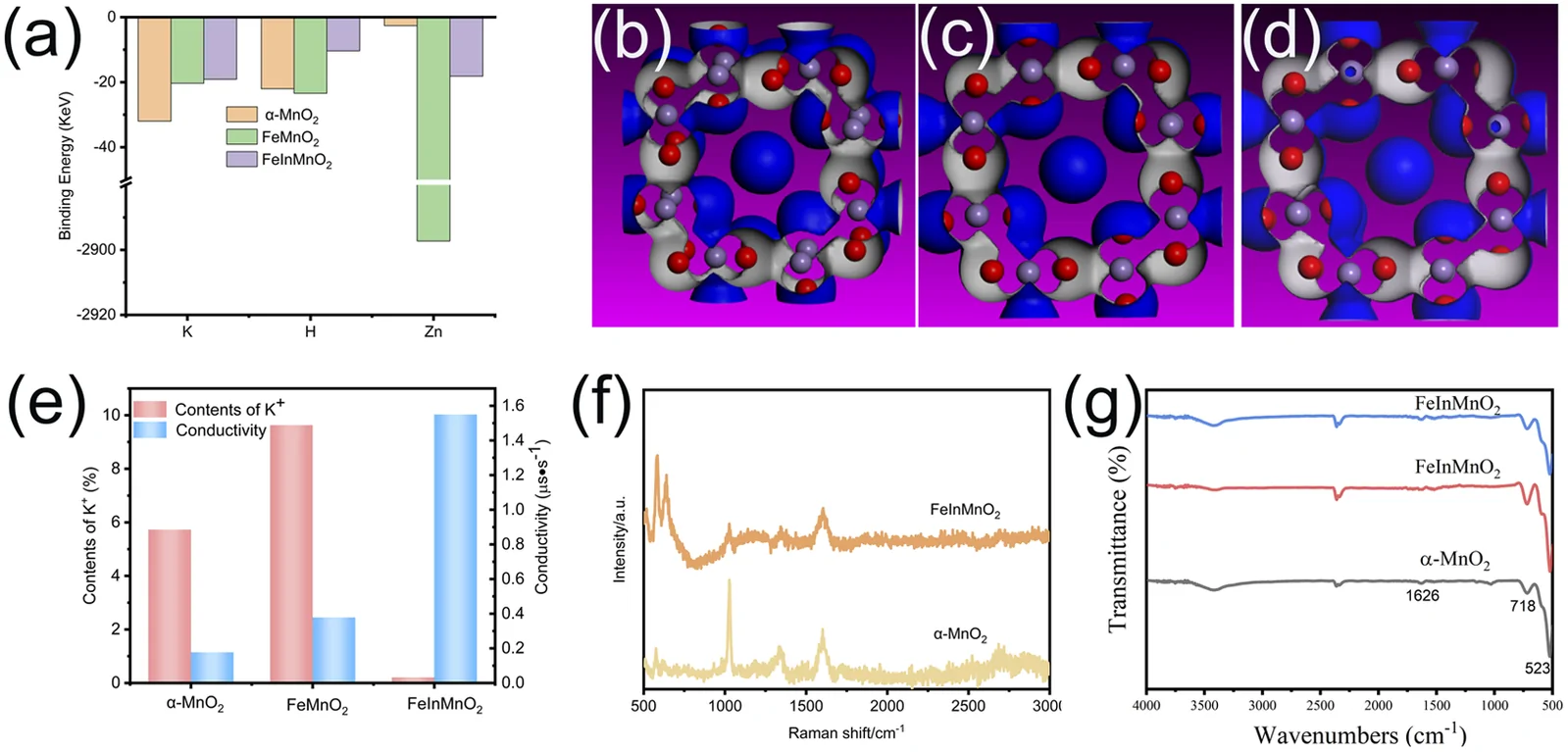
(a) Binding energy of α-MnO_2_, FeMnO_2_ and FeInMnO_2_. (b–d) Electron cloud density of α-MnO_2_, FeMnO_2_ and FeInMnO_2_. (e) ICP-MS analysis of the K^+^ content in the solid samples in α-MnO_2_, FeMnO_2_ and FeInMnO_2_ and conductivity of α-MnO_2_, FeMnO_2_ and FeInMnO_2_. (f) Raman spectra of α-MnO_2_, FeMnO_2_ and FeInMnO_2_. (g) FT-IR spectrum of α-MnO_2_, FeMnO_2_ and FeInMnO_2_.

### 3.2 Electrochemical measurements of three materials

The electrochemical measurements of α-MnO_2,_ FeMnO_2_ and FeInMnO_2_ as cathode materials with Zn to form a cell were performed in Fig. 5a. Fig. 5b displays the cyclic voltammetry (CV) curves of α-MnO_2_, FeMnO_2_ and FeInMnO_2_ cathodes at a scan rate of 0.1 mV s^−1^. In the cathodic scan, three samples possess two peaks at 1.2 and 1.3 V. This can be explained to the intercalation of Zn^2+^. In the anodic scan, the peaks at 1.60 V are attributed to the cations extraction process accompanied by the oxidation of Mn to higher the valence states.^25^ The galvanostatic charge/discharge (GCD) curves of α-MnO_2_, FeMnO_2_ and FeInMnO_2_ cathodes at the first three cycles under the current densities of 0.6 A g^−1^ are displayed in Fig. S4. Compared to Zn/Zn^2+^, the cathode material showed reduction and oxidation plateaus at 1300 mV and 1500 mV for the 3rd discharge and charge cycles, respectively. It is in agreement with the CV result. Furthermore, the FeInMnO_2_ cathode possesses a higher capacity of 347.52 mAh g^−1^ than the α-MnO_2_ (109 mAh g^−1^) and FeMnO_2_ (85.56 mAh g^−1^) cathodes. This result shows FeInMnO_2_ has the best electrochemical performances, which are attributed to In ions doped into manganese dioxide crystals, more active sites occupied by K^+^ ions are released. So more Zn^2+^ ions embedded/de-embedded in the tunnels of manganese dioxide in a fast way, and the electrode polarisation decreased. This mainly accounts for the facilitated ion (de)intercalation kinetics and the reduced electrode polarization observed for FeInMnO_2_, rather than for the absolute value of the capacity. The high specific capacities of 347.5 mAh g^−1^ exceed the one-electron Mn^4+^/Mn^3+^ limit expected for exclusive Zn^2+^ insertion/extraction, indicating that the charge storage is not due solely to Zn^2+^ (de)intercalation. It possibly involves a hybrid mechanism involving H^+^/Zn^2+^ co-insertion into the tunnels together with a reversible Mn^2+^ dissolution/deposition contribution enabled by the MnSO_4_ additive and a minor contribution from Fe^3+^/Fe^2+^ redox. Deconvoluting these contributions quantitatively dedicated *operando* studies, which we plan to addressed in a follow-up work. In short, the Fe–In co-doped into α-MnO_2_ as cathode materials can improve the electrochemical performance of aqueous Zn-ion battery (ZIBs). To further investigate the effects of Fe–In co-doping on the cycling performance of the battery, rate performance of aqueous ZIBs is evaluated at different current densities (Fig. 5c). As the current rates increase in the order of 0.2, 0.3, 0.4, 0.5, 0.6C, specific capacities of 437, 332, 310, 259, 227 and 200 mAh g^−1^ can be obtained for FeInMnO_2_ cathode. During an activation process, the specific capacity at the current rates of 0.2C displays a gradual increase from 280 mAh g^−1^ at the first time to 400 mAh g^−1^ at the tenth time. At the current rates of 0.3, 0.4, 0.5 and 0.6C, the discharge specific capacity gradually decreases to 332, 310, 259 and 200 mAh g^−1^. When it is returned to 0.2C again, the discharge specific capacity increases back to 437 mAh g^−1^. This result indicates that the FeInMnO_2_ cathode has excellent reversibility. However, for α-MnO_2_ electrode, a specific capacity of 170 mAh g^−1^ at the first charge/discharge is displayed, and with the cycling, the specific capacity was basically stable and unchanged at the current rates of 0.2C. At the same time, when the rate was increased to 0.3, 0.4, 0.5, and 0.6C, the specific capacities were 120, 100, 90, and 80 mAh g^−1^. This demonstrates the superior rate performance of FeInMnO_2_ cathode due to the enhanced conductivity. Besides, the cycle stability is also displayed in Fig. S4. Surprisingly, a specific capacity of 155.18 mAh g^−1^ is maintained after 10 000 cycles, which corresponds to a capacity retention rate of 94%. This should be attributed to the enhanced stability of the tunnel structure of α-MnO_2_. Fig. 5d–f and S5 display various capacity contribution ratios of α-MnO_2_, FeMnO_2_ and FeInMnO_2_ at different scan rates. For three materials, the ratio of the capacitance contribution increases with increasing scan rate. Interestingly, α-MnO_2_ has the lowest capacitance contribution ratio. The contribution of the capacitance of FeInMnO_2_ increases from 16.54% at 1 mV s^−1^ to 61.36% at 10 mV s^−1^. This arises from the higher conductivity and lower ion diffusion resistivity of FeInMnO_2_. We conducted galvanostatic intermittent titration technique (GITT) measurements to further estimate the diffusion coefficient of Zn^2+^ ions (*D*_Zn_^2+^) and the excellent performance of FeInMnO_2_. The detailed notes and parts of GITT profiles at charge and discharge platforms are displayed in Fig. S6. As expected, FeInMnO_2_ has a satisfactory ion diffusion coefficient of 10^−14^–10^−10^ cm^2^ s^−1^.^26^

**Fig. 5:**
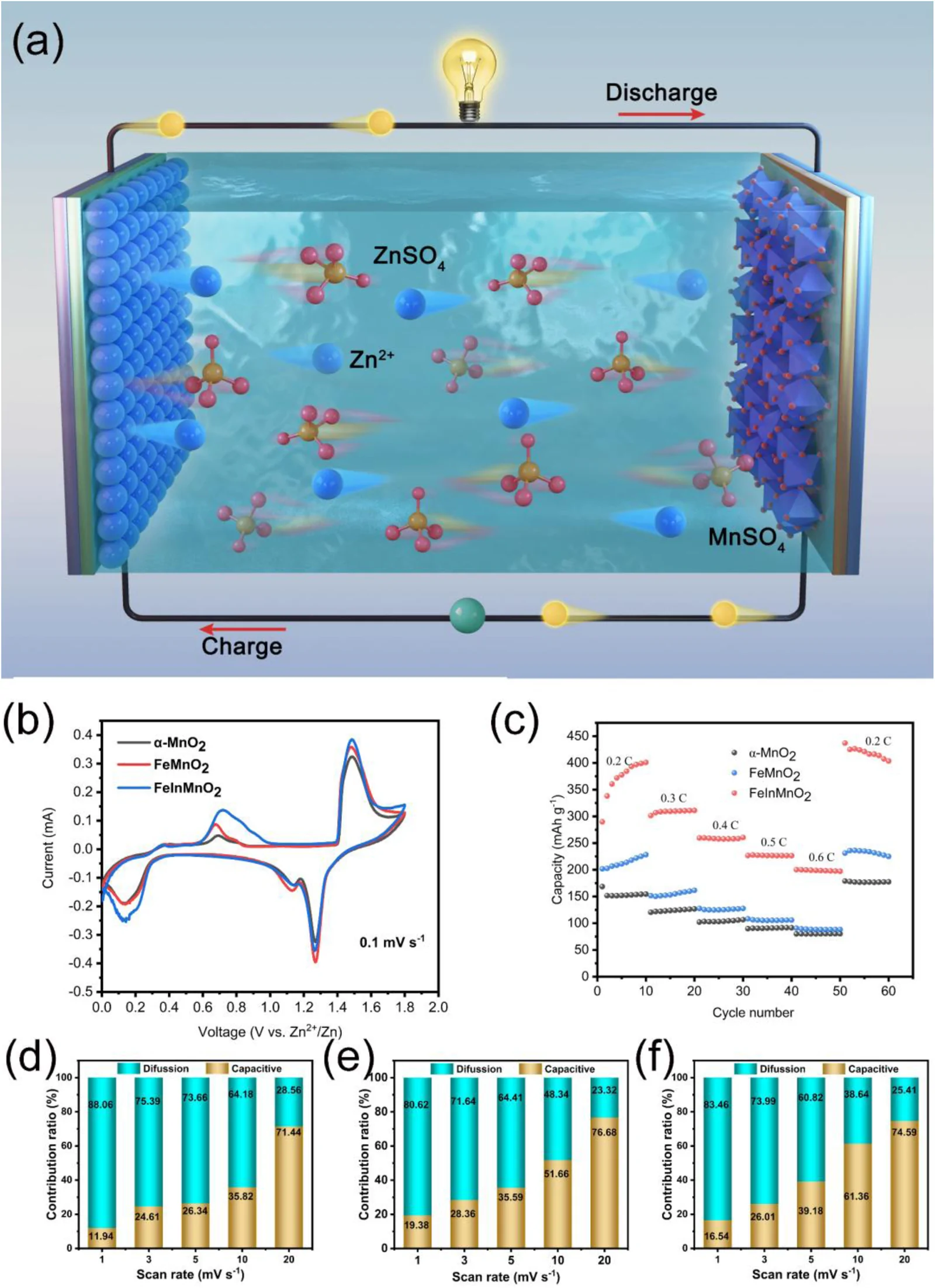
(a) Mechanistic picture of the α-MnO_2_, FeMnO_2_ and FeInMnO_2_ cathode in aqueous Zn-ion battery. (b) Cyclic voltammetry curves of α-MnO_2_, FeMnO_2_ and FeInMnO_2_ at a scan rate of 0.1 mV s^−1^. (c) Rate performance of α-MnO_2_, FeMnO_2_ and FeInMnO_2_. (d and e) The capacitive contribution ratio at different scan rates for the α-MnO_2_ (d), FeMnO_2_ (e) and FeInMnO_2_ (f).

## 4 Conclusion

In summary, heavy In doping into α-MnO_2_ mediated by β-FeOOH is achieved in this work. The content of In is significantly increased to 0.81% when iron is added as an auxiliary species. It can influence the intrinsic properties of α-MnO_2_ by changing the electronic structure. The binding energy with K^+^ ion and the K^+^ content of Fe–In co-doped α-MnO_2_ (FeInMnO_2_) is significantly reduced. Moreover, the conductivity is increased from 0.177 µS cm^−1^ to 1.55 µS cm^−1^. Due to the release of more active sites, the FeInMnO_2_ cathode has excellent electrochemical performance in zinc ion battery, such as better ion diffusion, reversibility, and long cycle stability. It opens a new avenue for the application of α-MnO_2_ in energy science.

## Author contributions

Hongmei Zhao: writing – review & editing, writing – original draft, visualization, validation, methodology, investigation, formal analysis, data curation. Song Li: writing – original draft, visualization, validation conceptualization. Jing Wang: methodology, investigation. Wai-Yeung Wong: writing – review & editing, supervision, resources, project administration, funding acquisition. Pengfei Liu: methodology, investigation. Jing Li: supervision, resources, project administration, funding acquisition. Junjie He: writing – review & editing, supervision, project administration, funding acquisition, conceptualization.

## Conflicts of interest

The authors declare that they have no conflict of interest.

## Supplementary Material

RA-OLF-D6RA07481B-s001

## Data Availability

Data will be made available on request. The authors have cited additional references within the supplementary information (SI). Supplementary information (SI) is available. See DOI: https://doi.org/10.1039/d6ra07481b.
