# Supplementary material for: β-FeOOH mediated heavy indium doping to release the tunnel space in α-MnO2
Source: RSC Adv. 2026 Nov 6. Online ahead of print. doi: 10.1039/d6ra07481b (PMC13741153; doi:10.1039/d6ra07481b)
Supplement: Supplementary file 1 [file RA-OLF-D6RA07481B-s001.pdf]

Supplementary Information (SI) for RSC Advances.
This journal is © The Royal Society of Chemistry

Supporting Information

β-FeOOH mediated heavy indium doping to release the tunnel space in α-MnO2

Hongmei Zhao[a,+], Song Li[a,+], Jing Wang[a], Wai-Yeung Wong[b,*], Pengfei Liu[c], Jing Li[a,*] and Junjie He[a,*]

[a] *Department of Science and Technology, Yunnan Agricultural University, Kunming 650201, China*

[b] *Department of Applied Biology and Chemical Technology and Research Institute for Smart Energy., The Hong Kong Polytechnic University, Hung Hom, Hong Kong, China*

[c] *Spallation Neutron Source Science Center, Institute of High Energy Physics, Chinese Academy of Sciences, Dongguan 523803, China*

[*] *Corresponding author.*

E-mail address: junjiehe@ynau.edu.cn (J. He); lijing69@ynau.edu.cn (J. Li); wai-yeung.wong@polyu.edu.hk (W.-Y. Wong)

[+] These authors contributed equally to this work.

1. Theoretical calulations

Material studio program was used to carry out the theoretical calculations. The Perdew-Burke-Ernzerhof for solid (PBESol) exchange-correlation functions of the gener-alized gradient approximation (GGA) was applied for the treatment of electronic-exchange correlation potential. The DFT+U approach was used to calculate the total energy and density of state of the electrons. The convergence criteria of the maximum energy change, the maximum displacement, and the maximum stress were set as 2×10^{-5} eV/atom, 2×10^{-3} Å, and 0.1GPa, accordingly. Spin polarization was considered in all calculations. For MnO_2 unitcell optimization, 1×1×1 k-point samplings were used. Based on the initial MnO_2 unitcell (tetragonal; space group I4/m; a=9.777(2) and c=2.8548(5) Å, it was expanded to 2×1×1 supercell, and substituted one Mn atom with Mx (Fe or In) to get the doped supercells. Binding energy (E_b) is defined as follows,

$$E_b = E_{cell} - E_{Mn} - E_O - E_K - E_X \quad (3)$$

where E_{cell} is the total energy of the α-MnO_2 supercell, and E_{Mn}, E_O, E_{Na} and E_x are the energy of Mn, O and X (Fe or In), respectively.

2. Experimental data

2.1 XRD analysis of α-MnO_2、$FeMnO_2$、$FeInMnO_2$

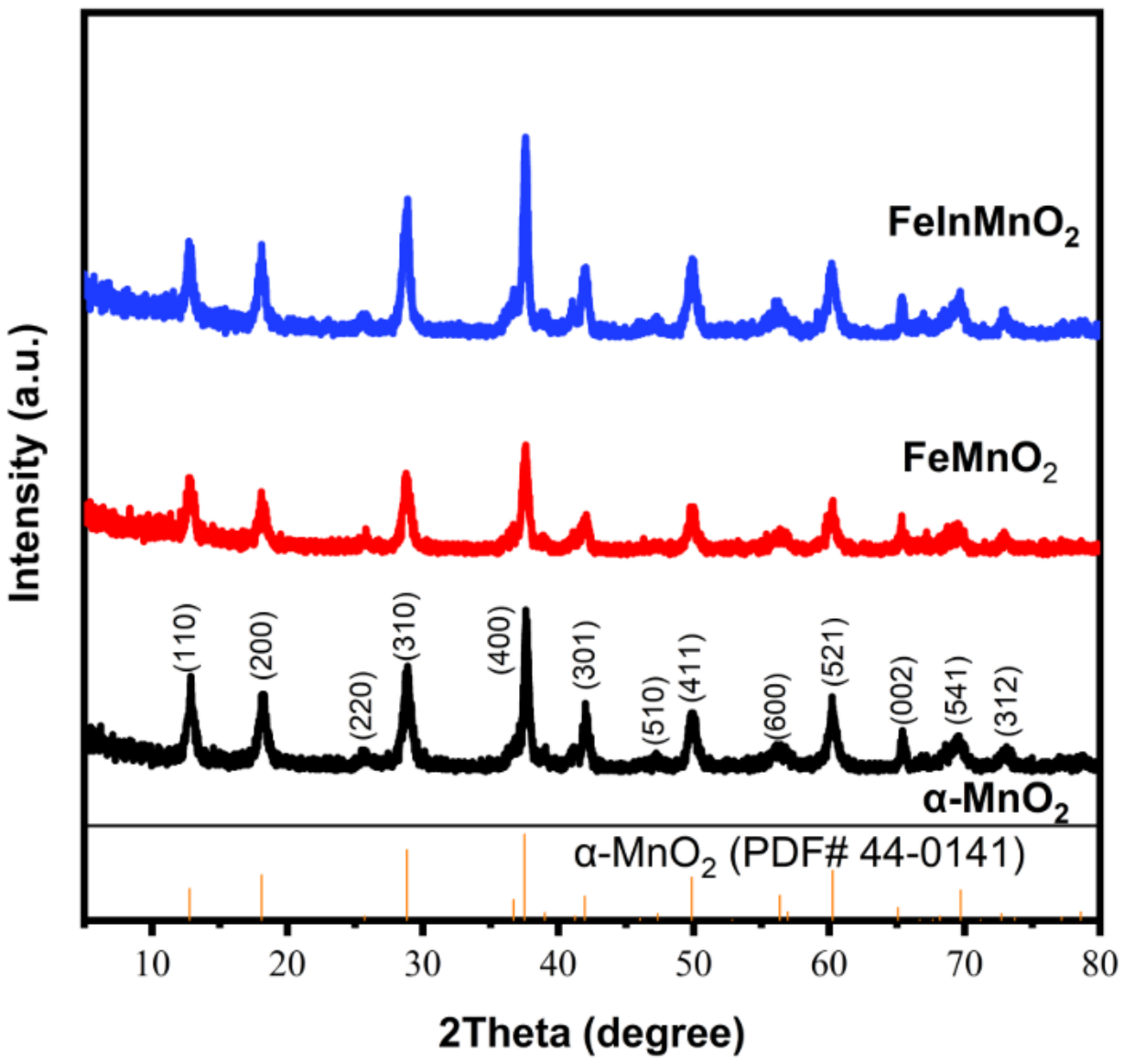

Figure S1. XRD patterns of α-MnO_2、$FeMnO_2$、$FeInMnO_2$

Table S1 Refined lattice parameters

Phase No.	Free （a）		Fe (b)		Fe In (d)	
Space Group	I 4/m		I 4/m		I 4/m	
Cell parameters	a(Å)	9.84560(72)	a(Å)	9.83992(54)	a(Å)	9.8373(18)
	b(Å)	9.84560(72)	b(Å)	9.83992(54)	b(Å)	9.8373(18)
	c(Å)	2.86059(31)	c(Å)	2.86105(21)	c(Å)	2.86276(58)
	α(°)	90	α(°)	90	α(°)	90
	□(°)	90	□(°)	90	□(°)	90
	□(°)	90	γ(°)	90	γ(°)	90
	Volume-Å3	277.293(50)	Volume-Å3	277.019(36)	Volume-Å3	277.04(12)

2.2 BET analysis of α-MnO_2、$FeMnO_2$、$FeInMnO_2$

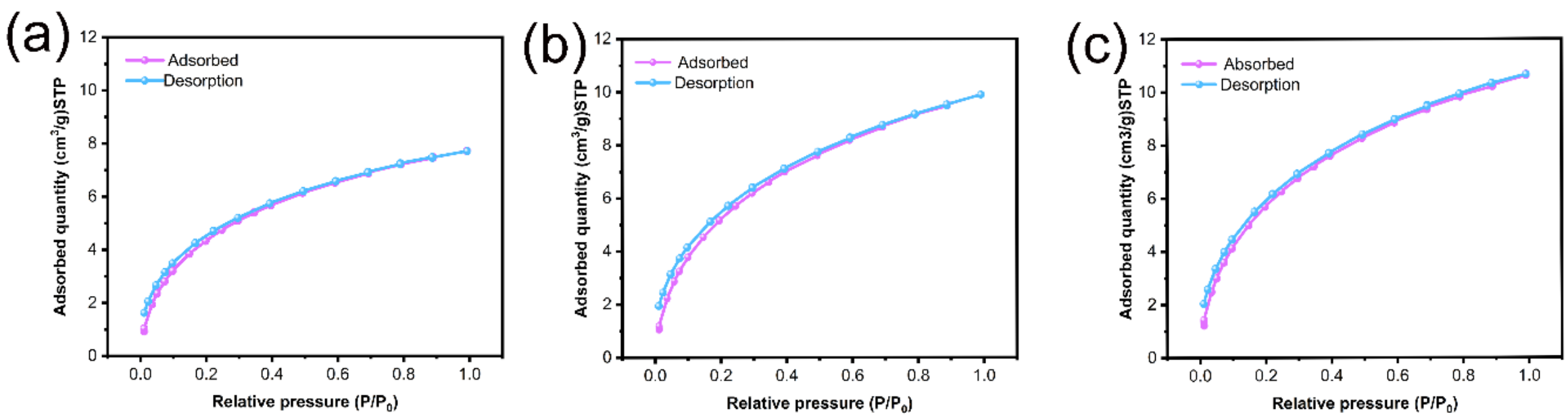

Figure S2. The BET map of α-MnO_2 (a)、$FeMnO_2$ (b)、$FeInMnO_2$(c)

Table S2 BET test parameters

Sample	S_{BET} [m² g⁻¹][a]	V_{tot} [cm³ g⁻¹][b]	D_{BET} [nm][c]	S_{BJH} [m² g⁻¹][d]	V_{BJH} [cm³ g⁻¹][e]	D_{BJH} [nm][f]
α-MnO_2	17.3036	0.0119	2.7509	17.4994	0.0118	2.6972
$FeMnO_2$	21.4527	0.0153	2.8528	22.9940	0.0158	2.7485
$FeInMnO_2$	23.2108	0.0165	2.8435	25.2639	0.0172	2.7233

[a] *Surface area is calculated by Brunauer–Emmett–Teller (BET) method.*

[b] *Total pore volume is obtained at P/P_o = 0.99.*

[c] *Average pore width is calculated from Brunauer–Emmett–Teller (BET) method.*

[d] *BJH surface area is calculated from Barrett–Joyner–Halenda (BJH) method.*

[e] *BJH volume is calculated from Barrett–Joyner–Halenda (BJH) method.*

[f] *Average pore width is calculated from Barrett–Joyner–Halenda (BJH) method.*

2.3 The total binding energies (E_b)

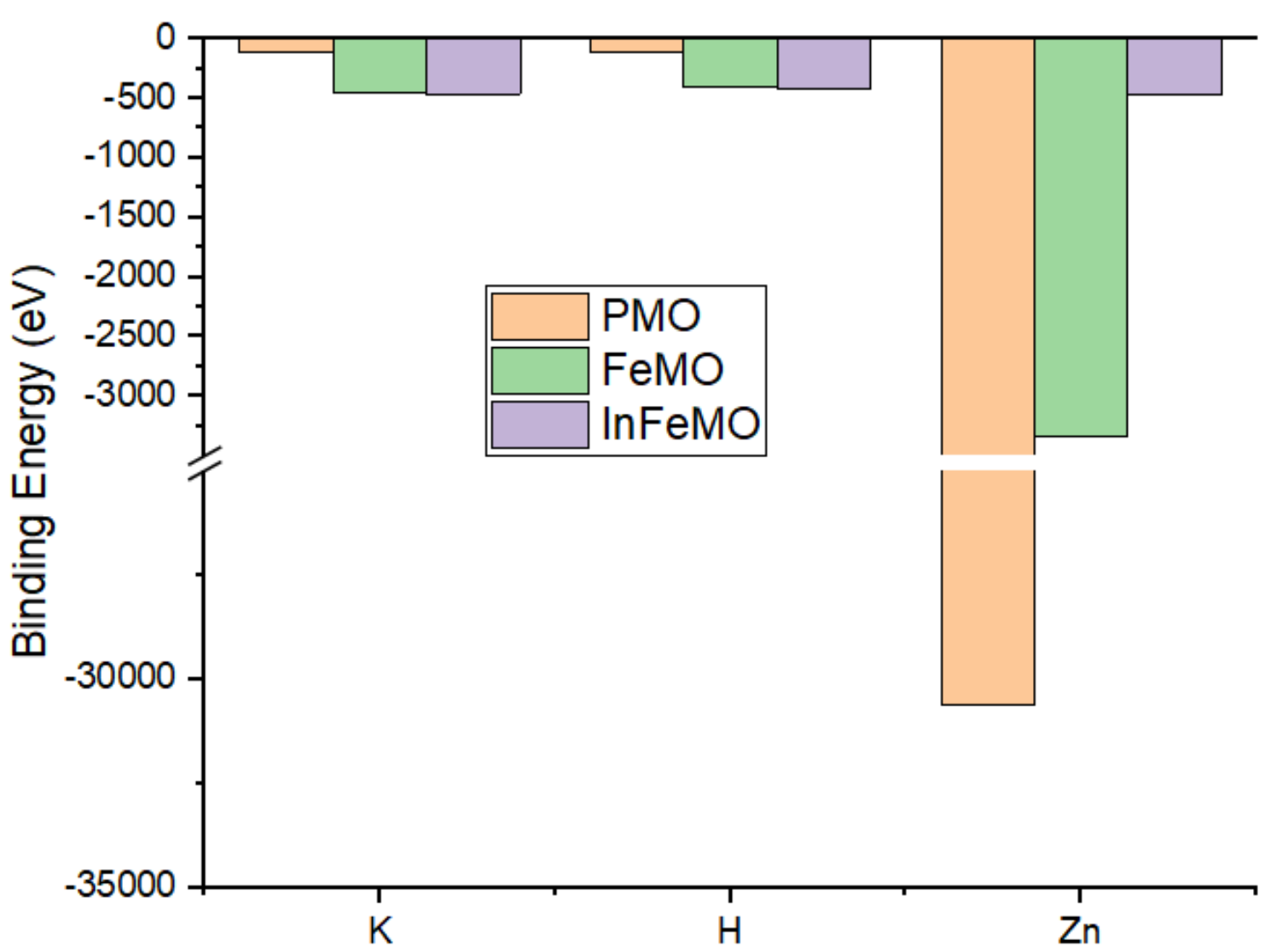

Figure S3. The total binding energies (E_b) of α-MnO_2、$FeMnO_2$、$FeInMnO_2$

2.4 Electrochemical analysis of α-MnO_2、$FeMnO_2$、$FeInMnO_2$

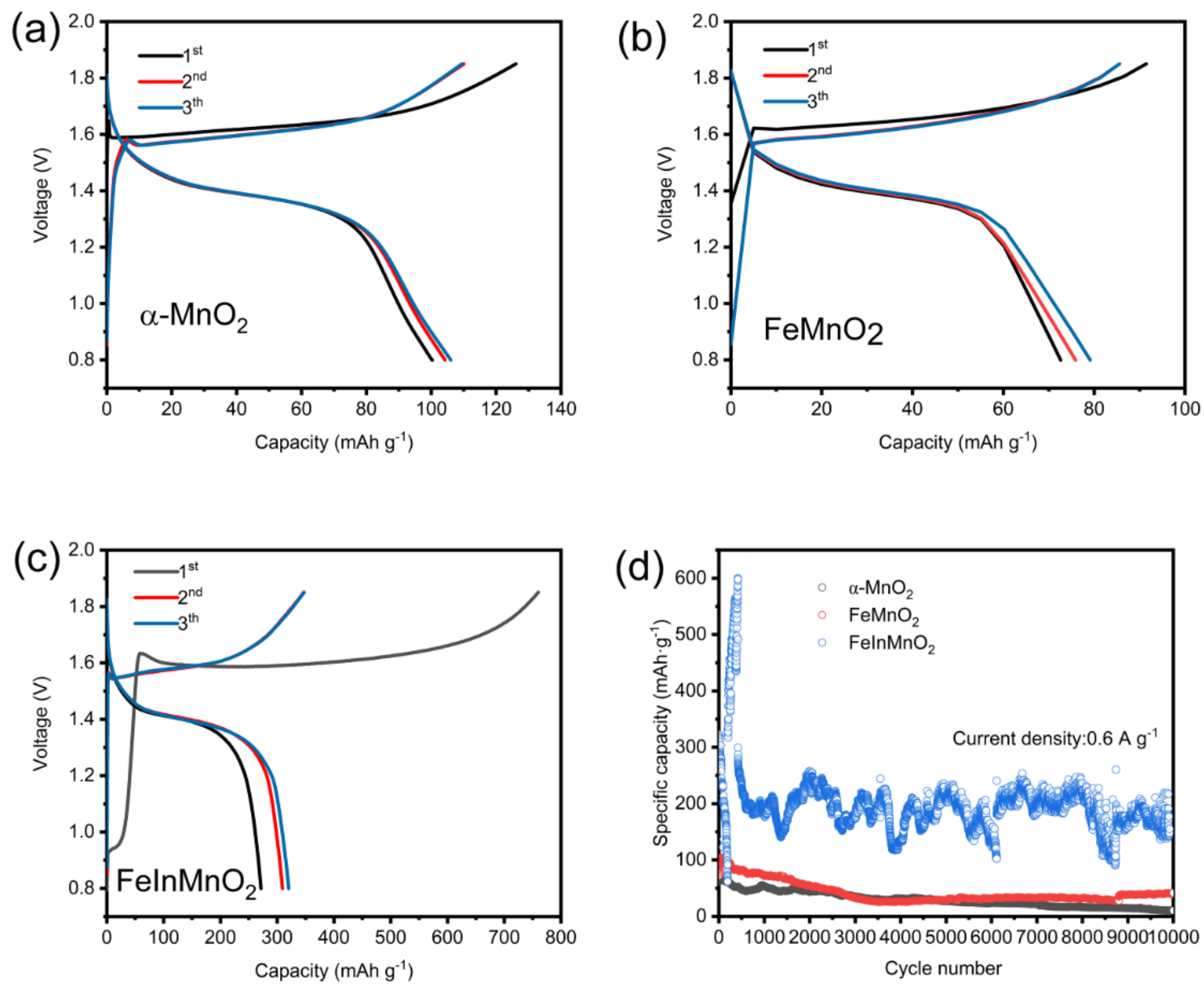

Figure S4. (a) Galvanostatic charge/discharge curves of α-MnO_2 at a current density of 0.6 A g^{-1}. (b) Galvanostatic charge/discharge curves of $FeMnO_2$ at a current density of 0.6 A g^{-1}. (c) Galvanostatic charge/discharge curves of $FeInMnO_2$ at a current density of 0.6 A g^{-1}. (d) Long-term cycling stability of of α-MnO_2, $FeMnO_2$, $FeInMnO_2$ at 0.6 A g^{-1}

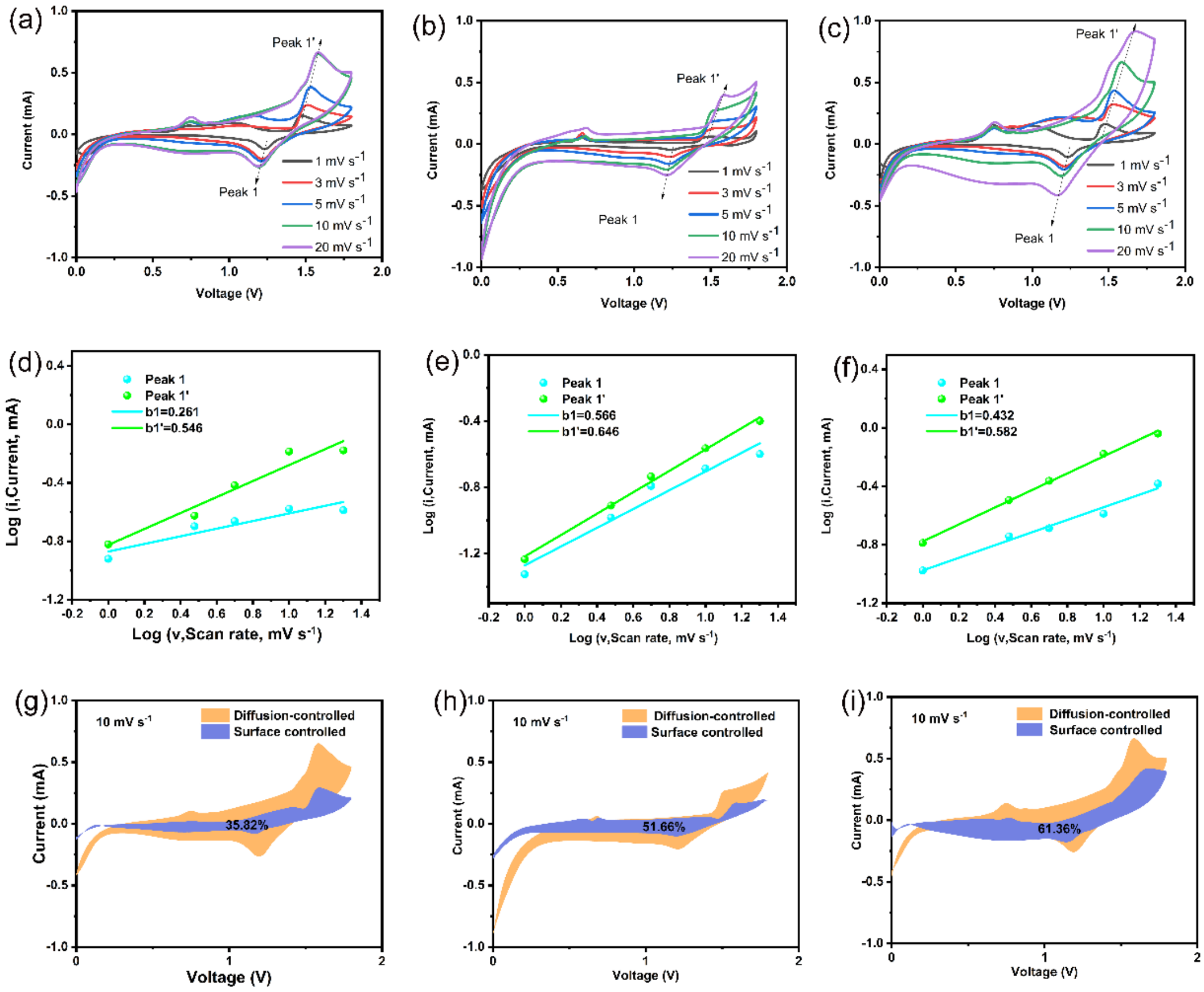

Figure S5. CV curves of α-MnO$_2$ (a)、FeMnO$_2$ (b) and FeInMnO$_2$ (c)cathode at various scan rates; (e-f) Determination of the b value according to log *i* and log *v* plots at specific peak currents; (g-i) CV curve of the α-MnO$_2$ 、 FeMnO$_2$ and FeInMnO$_2$ electrode with the shadowed area representing the surface capacitive contribution.

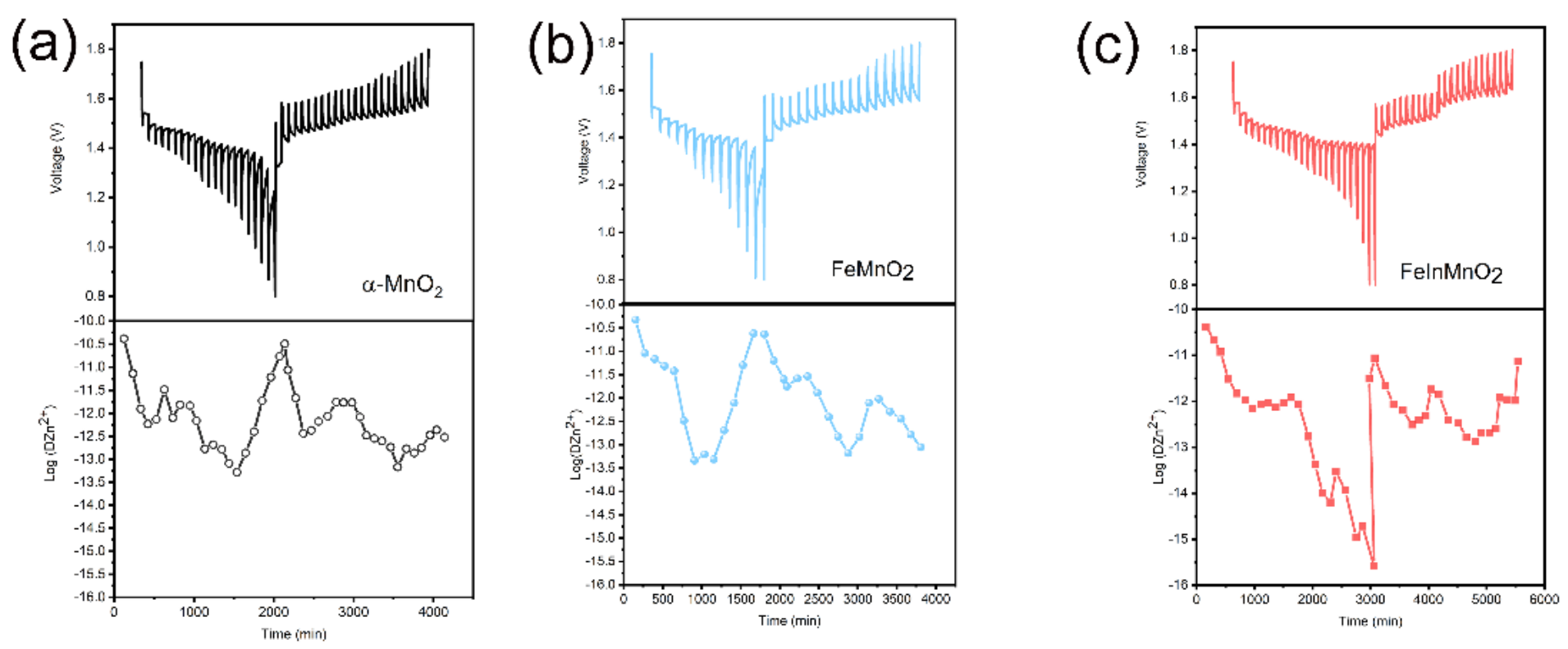

Figure S6. The charge/discharge GITT profiles of (a) α-MnO_2, (b) $FeMnO_2$ and (c) $FeInMnO_2$ and the corresponding diffusive coefficients (cm^2 s^{-1}) are presented.
